# The meniscofemoral ligaments do not contribute to restriction of posterior tibial translation: A robotic biomechanical analysis

**DOI:** 10.1002/ksa.12812

**Published:** 2025-08-05

**Authors:** Lucas Palma Kries, Wenke Liu, Michael J. Raschke, Alina Albert, Christian Peez, Elmar Herbst, Christoph Kittl, Adrian Deichsel

**Affiliations:** ^1^ Department of Trauma, Hand and Reconstructive Surgery University Hospital Münster Münster Germany

**Keywords:** knee, PCL, posterior cruciate ligament, MFL, restraint, PTT

## Abstract

**Purpose:**

The aim of this study was to quantify the contribution of the meniscofemoral ligaments (MFLs) on restraining a posterior tibial translation (PTT) in the human knee joint.

**Methods:**

Sixteen human cadaveric knee joints were tested in a robotic test setup with six degrees of freedom. Knees with no MFL, one MFL and two MFLs were included. Knee joints without MFLs were excluded from the statistical analysis regarding the influence of MFLs on PTT. The knees were tested in a displacement‐controlled protocol, which replayed the native kinematics of a force controlled test protocol with PTT at 89 N in neutral tibial rotation, PTT in 5 Nm internal rotation, PTT in 5 Nm external rotation, while constantly measuring the force. The principle of superposition was used to determine the contribution of each cut structure (in‐situ forces) to restraint of the performed movements. First, the anterior and posterior MFL (aMFL/pMFL) were randomly cut, followed by the posterior cruciate ligament (PCL).

**Results:**

Neither the aMFL, nor the pMFL showed a significant contribution to the restriction of PTT in the PCL‐intact knee neither in 0°, 30°, 60° or 90° of flexion in neutral, internal, or external rotation (*p* > 0.05). The PCL showed a significant contribution to the knee joint restraining PTT in 0°, 30°, 60° and 90° flexion as well as in all rotation states (all *p* < 0.05). A contribution of the PCL restraining PTT of 28% ± 14% in 0° flexion, 53% ± 21% in 30° flexion, 61% ± 20% in 60° flexion and 54% ± 16% in 90° knee flexion was measured in neutral rotation.

**Conclusion:**

The MFLs do not contribute to restriction of a PTT in any flexion angle, while the PCL acts as the primary restraint against PTT from 0°–90° knee flexion. This effect was seen in neutral rotation as well as in tibial internal and external rotation. This study indicates that a dissection of the MFLs to gain access to the PCL during reconstruction surgery does not destabilise the knee.

**Level of Evidence:**

N/A.

**Abbreviations**
aMFL: anterior meniscofemoral ligament
ATT: anterior tibial translation
DOF: degrees of freedom
MFL: meniscofemoral ligament
PCL: posterior cruciate ligament
pMFL: posterior meniscofemoral ligament
PTT: posterior tibial translation

## INTRODUCTION

The primary restraint of posterior tibial translation (PTT) is the posterior cruciate ligament (PCL) [2, 23, 28]. Besides the PCL different authors identified the meniscofemoral ligaments (MFL) to provide significant contribution to the knee resisting a PTT [19, 24].

The MFLs attach the posterior horn of the lateral meniscus to the medial femoral condyle in addition to the posterior root. The literature describes the presence of an anterior meniscofemoral ligament (aMFL) in 40.1% [6], and a posterior meniscofemoral ligament (pMFL) in 60.9% of knee joints [6]. The MFLs, known as the ligaments of Humphrey (aMFL) and Wrisberg (pMFL) pass anteriorly and posteriorly to the posterior cruciate ligament (PCL) and attach to the medial condyle proximal to the posteromedial bundle of the PCL [3].

In different biomechanical studies a stabilising function of the MFLs to the lateral meniscus could be shown as the femorotibial contact pressure significantly increased only after combined dissection of the posterior root of the lateral meniscus and the MFLs [11, 12]. Additional to this function different authors postulated the MFLs to contribute significantly as a secondary restraint to PTT [19, 25]. Supporting this hypothesis, in a clinical observation of patients with a ruptured PCL a correlation between a reduced posterior drawer with the tibia in internal rotation and intact MFLs was found [4]. Biomechanical studies however showed conflicting results regarding the stabilising function of the MFLs [19, 25, 29]. These studies showed major methodical differences, so that there remains uncertainty about the role of the MFLs in restraining PTT. If there exists a significant contribution of the MFLs to restraining PTT, care should be taken during PCL reconstruction surgery to not damage the MFLs. Thus, the aim of this study was to biomechanically quantify the influence of the MFLs in restraining a PTT using a robotic test setup. Based on clinical experience, it was hypothesised that the MFLs do not significantly contribute to restraining a PTT.

## MATERIALS AND METHODS

16 unpaired cadaveric knee specimens (mean age 67.3 ± 7.4 years, 11 male and 5 female) without history of prior knee surgery, or injury, were obtained from an international tissue bank (MedCure). The specimens were visually inspected for high‐grade osteoarthritis or meniscal injury. Furthermore, ligamentous instability testing was performed by the investigator. Specimens were excluded if pathologies were present. A prior evaluation for the presence of MFLs was not carried out. Knee joints without MFLs were not included in the statistical analysis regarding the influence of MFLs on PTT.

Ethical permission for the study was obtained from the institutional review board of the university of Münster (IRB reference number 2023‐407‐f‐S).

The knee joints were stored frozen at −20°C in plastic bags without chemical fixation. Before testing the specimens were thawed for 24 h at room temperature. To prepare the knee joints for the experiments skin and subcutaneous tissue were resected, leaving the remaining soft tissue intact. 12 cm above and below the joint line, femur and tibia were cemented in aluminium cylinders using three‐component polyurethane bone cement (RenCast®; Gößl & Pfaff). The fibula was then cut 10 cm below the joint line and transfixed with a 3.5 mm cortical screw to the tibia [30]. Specimens were wrapped in wet tissue papers to prevent drying.

### Robotic test setup

The biomechanical testing was performed in a validated test setup consisting of an industrial robot (KR 60‐3, KUKA Robotics) [7, 8, 9, 21]. The test setup allows the manipulation of the knee joint in all six degrees of freedom (6‐DOF). The robot can carry a maximum load of 60 kg and can be moved position‐controlled within its radius of action with an accuracy of ±60 µm. A force‐controlled movement can be executed with an accuracy of ±0.25 N and ±0.05 Nm respectively [24].

The test setup was driven by the custom software simVITRO (Cleveland Clinic BioRobotics Lab), which optimises the robot test setup for the simulation and acquisition of knee joint kinematics. To move the specimens precisely and physiologically the knee joint coordinate system was defined according to the descriptions by Grood and Suntay [17]. This was achieved by digitising landmarks on the femur and tibia, using a tactile measuring arm (Absolute Arm 8320‐7; Hexagon Metrology GmbH) with an accuracy of ±0.05 mm. In this setup translations and rotations of the tibia in relation to the femur and the corresponding forces and torques in all six degrees of freedom are recorded continuously.

### Biomechanical testing

Each specimen was extended and flexed ten times to obtain tissue hysteresis [26]. By minimising all pressures and torques acting on each knee in 0° of flexion using custom software, the starting point of each knee was identified. Next, each knee was flexed from 0° of flexion to 90° of flexion, reducing forces and torques in all axes aside from the flexion‐extension axis, to determine the 'passive path' of the knee. To ensure contact between femur and tibia an axial compression force of 50 N was applied during the passive path.

The contribution of the MFLs and PCL to restricting anterior tibial translation (ATT) and PTT was tested via superposition. First, a force‐controlled protocol (recording displacements in response to given forces/torques) with the application of 89 N anterior tibial translation (ATT), followed by 89 N PTT was performed in 0°, 30°, 60° and 90° knee flexion while keeping an axial compression of 200 N. This simulates the force applied by a KT‐2000 arthrometer [5]. Then these movements were repeated with the knees moved in internal and external rotation with 5 Nm, simulating a posteromedial or posterolateral drawer test, to control whether different rotational angles alter the involvement of the MFLs in restraining a PTT [4, 22].

The motion obtained of these movements was then transferred into a displacement‐controlled protocol, which repeated the native knee kinematics, and allowed to measure the forces needed to reach the target position. After each cutting step the reduction of force needed to perform the desired movement indicated the contribution of the ligaments restraining PTT (in situ forces), according to the principle of superposition [13, 31].

### Sequential cutting protocol

**Figure 1:**
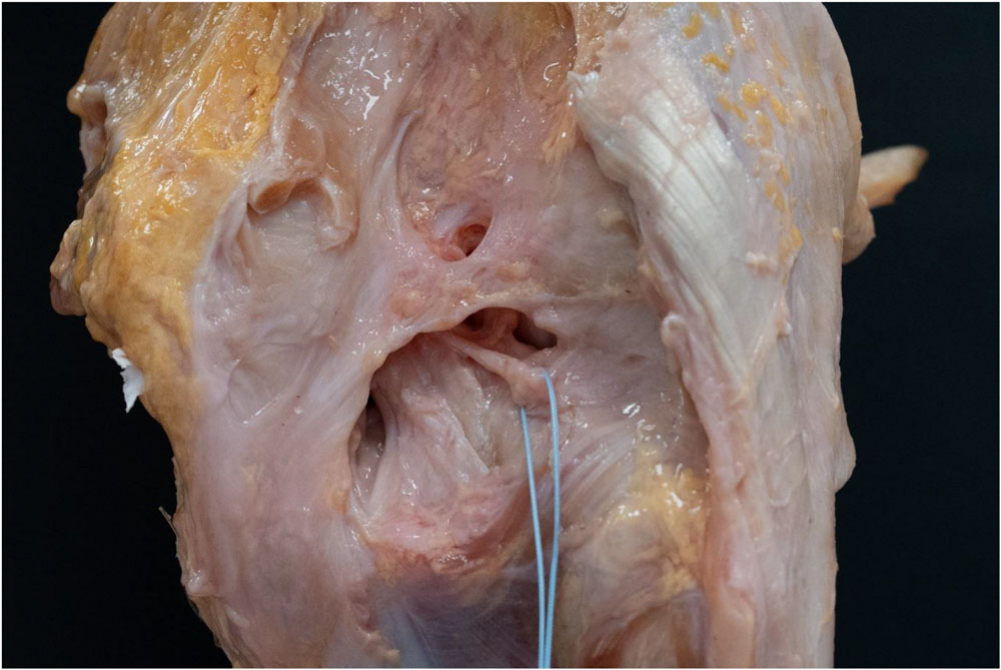
Posterior view of a human right knee after establishing a posterior approach by longitudinally cutting the posterior joint capsule. The posterior meniscofemoral ligament is looped with a thread and runs posteriorly and parallelly to the posterior cruciate ligament.

**Figure 2:**
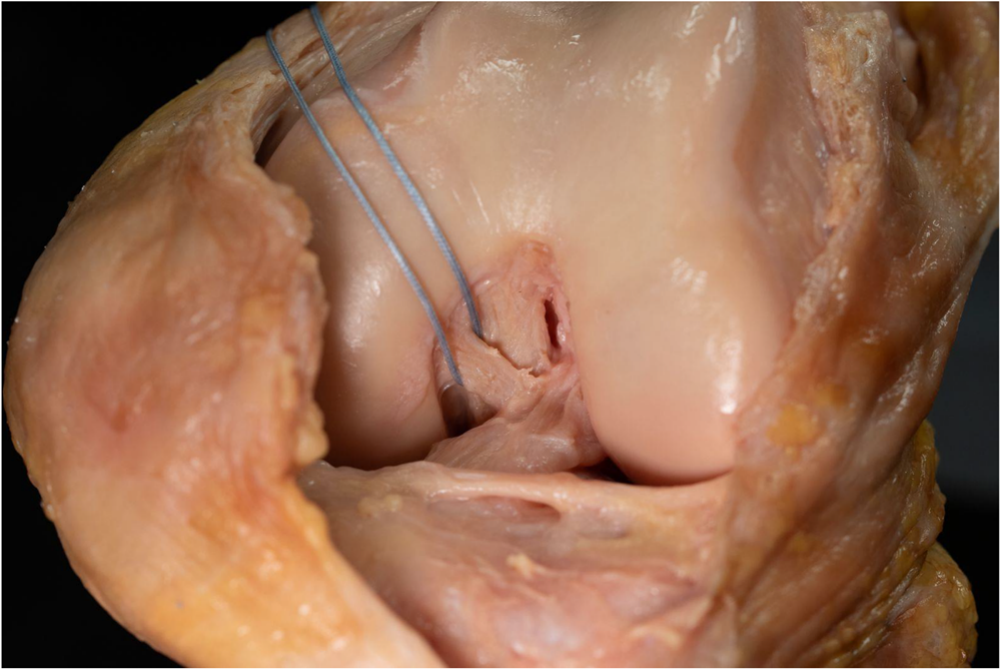
Anterior view of a human left knee after lateral parapatellar arthrotomy. The anterior meniscofemoral ligament is looped with a thread and runs between the anterior and the posterior cruciate ligament. The fibres are attached to the anterior lateral side of the medial femur.

First, the posterior joint capsule was split at the centre of the tibial width to establish view on the PCL and to verify the presence of a pMFL. The capsular incisions were closed prior to testing as there was shown that these closed incisions do not have an effect on laxity testing of the knee [14]. The cut was set between the superior border of the popliteus muscle and the oblique popliteal ligament. The pMFL was identified by the attachment to the lateral meniscus and the obliquity of the fibres in contrast to the steep fibre course of the PCL (Figure 1). Then a lateral parapatellar arthrotomy was performed to gain access to a potentially present aMFL. The identification of the aMFL again was based on the oblique fibre course compared to the straight course of the PCL fibres and the attachment to the lateral meniscus when followed distal (Figure 2).

If both MFLs were present, the aMFL and pMFL were openly cut in a randomised order, if one of the MFL was present it was cut after testing the native state. After testing the MFL‐deficient knee was completed, the PCL was cut, and the knee was tested again.

### Statistical analysis

The knee kinematics were extracted from the raw data of SimVitro using Matlab (version R2020a, MathWorks), and Excel (Microsoft). The statistical analysis was performed using PRISM (Version 10, GraphPad Software). The contribution of the different structures to restraining PTT is presented as a percentage of the total forces used in the movement to obtain the native kinematic (89 N). The comparison of the total contribution of the MFLs and the PCL in different flexion angles was performed utilising mixed linear models, to assess the main effects and interactions of each independent variable (cutting state in different flexion angles), with the dependent variable being the PTT. Post hoc pairwise comparisons with Dunnett's correction were used to compare contribution of each cutting state against the native state (0% loss of force). A *p*‐value less than 0.05 was deemed to identify significant differences. In a subgroup analysis multiple t‐tests were used to compare the contribution of the MFLs between knees with a single and both MFLs.

To determine the sample size necessary for the present study, an a‐priori power analysis was performed using G*Power (version 3.1) [10]. Based on previous studies investigating the influence of MFLs restraining PTT [19], a sample size of *n* = 13 was calculated to show a 10% contribution of a cutting step (assuming a standard deviation of 10%; effect size = 1), with a power of 90%, at the significance level of *p* < 0.05.

## RESULTS

### Presence of meniscofemoral ligaments

Of the 16 cadaveric knee specimens tested, an aMFL was present in 13 knees (81%) and a pMFL was found in 11 knees (69%). In 10 knees (63%) both MFLs were present. In two knees (13%) no MFL was found (Table 1). Therefore, only the native and the PCL‐deficient state were finally analysed in these two knee joints.

**Table 1:** Presence of the anterior meniscofemoral ligament (aMFL) and posterior meniscofemoral ligament (pMFL) in the tested knees.

State	*n*	%
Single ligament	4	25
Both MFLs	10	63
No MFL	2	13
Total aMFL	13	81
aMFL only	3	19
Total pMFL	11	69
pMFL only	1	6

### Restraints of PTT

**Figure 3:**
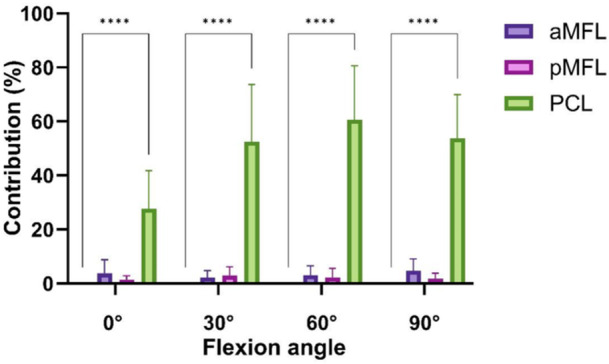
Contribution of the tested structures to restraining posterior tibial translation (in %) in neutral tibial rotation. ****p < 0.0001. aMFL, anterior meniscofemoral ligament; PCL, posterior cruciate ligament; pMFL, posterior meniscofemoral ligament.

In neutral tibial rotation the PCL contributed significantly to restraining PTT at 0° knee flexion with 28% ± 14%, at 30° knee flexion with 53% ± 21%, at 60° with 61% ± 20% and at 90° flexion with 54% ± 16% respectively (*p* < 0.05) (Figure 3). The aMFL and pMFL did not show a significant contribution to restraining PTT in all knee flexion angles and neutral tibial rotation (Table 2, *p* > 0.05).

**Table 2:** Contribution of the meniscofemoral ligaments to the force resisting posterior tibial translation.

Tibial rotation	Contribution of aMFL (% ± SD)			Contribution of pMFL (% ± SD)		
Flexion angle	NR	ER	IR	NR	ER	IR
0°	4 ± 5	4 ± 5	3 ± 5	1 ± 1	1 ± 1	0 ± 1
30°	2 ± 3	3 ± 4	2 ± 3	3 ± 3	2 ± 2	1 ± 3
60°	3 ± 3	2 ± 3	3 ± 5	2 ± 3	2 ± 2	2 ± 2
90°	5 ± 4	3 ± 4	3 ± 3	2 ± 2	2 ± 2	1 ± 2

*Note*: None of the contributions shown are significant (*p* > 0.05).

Abbreviations: aMFL, anterior meniscofemoral ligament; ER, external tibial rotation; IR, internal tibial rotation; NR, neutral tibial rotation; pMFL, posterior meniscofemoral ligament; SD, standard deviation.

**Figure 4:**
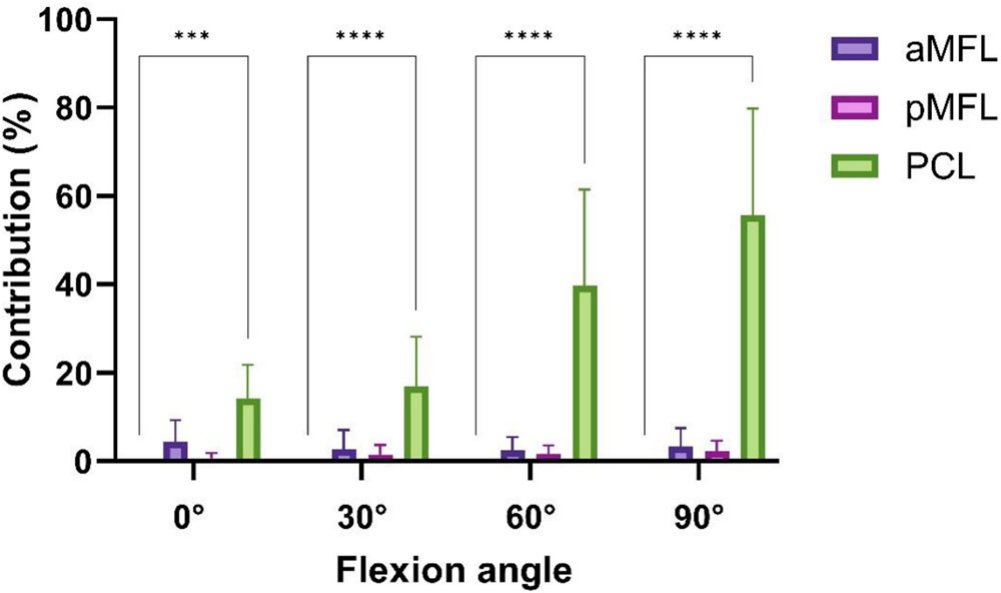
Contribution of the tested structures to restraining posterior tibial translation (in %) in external tibial rotation. ***p < 0.001; ****p < 0.0001. aMFL, anterior meniscofemoral ligament; PCL, posterior cruciate ligament; pMFL, posterior meniscofemoral ligament.

When tested in external tibial rotation the only structure tested providing significant resistance against PTT was the PCL. In 0° knee flexion it provided a contribution of 14% ± 7% against PTT. In 30° knee flexion it showed a contribution of 17 % ± 11%, in 60° knee flexion of 40% ± 22% and in 90° flexion angle of 56% ± 24% to the force restraining PTT (*p* < 0.05) (Figure 4). The aMFL and pMFL did not contribute significantly to restraining PTT in all knee flexion angles and external tibial rotation (Table 2, *p* > 0.05).

**Figure 5:**
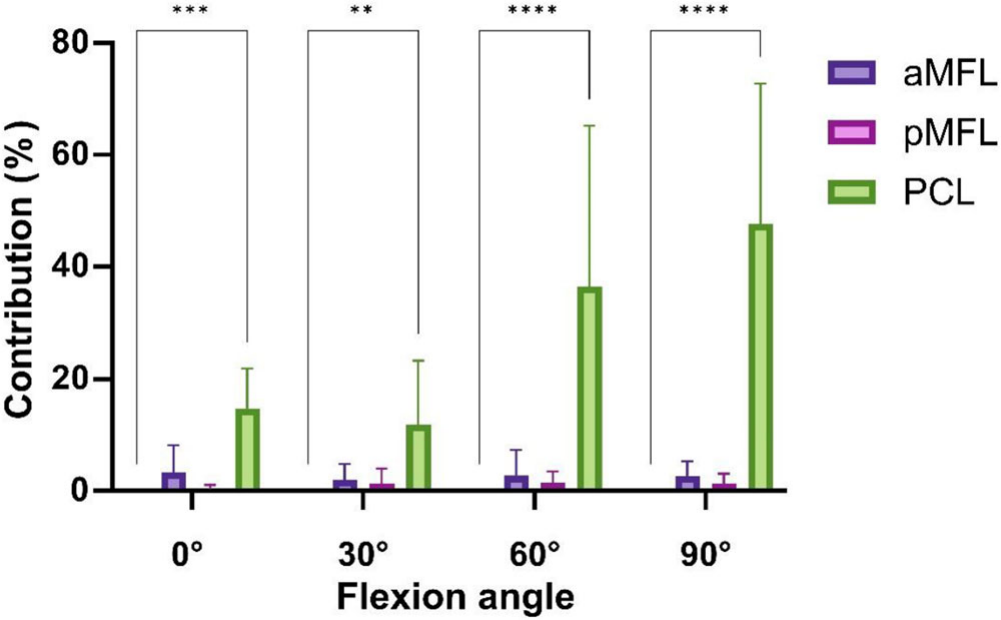
Contribution of the tested structures to restraining posterior tibial translation (in %) in internal tibial rotation. **p < 0.01; ***p < 0.001; ****p < 0.0001. aMFL, anterior meniscofemoral ligament; PCL, posterior cruciate ligament; pMFL, posterior meniscofemoral ligament.

In internal rotation the PCL also was the only structure which provided significant contribution to restraining PTT. In 0° knee flexion it contributed 15% ± 7% to the resistance to PTT. In 30° knee flexion we measured 12% ± 11%, at 60° knee flexion 36% ± 29% and at 90° flexion angle 48% ± 25% contribution against PTT (*p* < 0.05) (Figure 5). The aMFL as well as the pMFL did again not show significant contribution to restraining PTT in all knee flexion angles and internal tibial rotation (Table 2, *p* > 0.05).

A subgroup analysis showed no significant difference in the influence of MFLs between knee joints with a single or both MFLs in all flexion angles and tibial rotation states (*p* > 0.05).

## DISCUSSION

The most important finding of the present study was that the MFLs did not significantly contribute to restraining a PTT, in neutral rotation as well as in internal and external rotation. The primary restraint against PTT force was the PCL in all flexion angles. Furthermore, an increase in the involvement of the PCL in increasing degrees of flexion in the resistance of the knee joint to PTT was shown. This was evident in internal and external rotation, while in neutral tibial rotation the peak was shown at 60° knee flexion.

Various statements can be found in the literature on the involvement of the MFL in resistance to PTT. In a clinical study, a tightening of the MFLs in internal rotation and a following function as secondary restraint to a posterior drawer was proposed [4]. The observed influence may be due to posterolateral root lesions, which were not reported. The present study could not confirm this theory as there was no significant reduction of the force needed to reach the PTT of the intact knee after cutting of the MFLs.

A biomechanical study tested eight human knees to assess anterior‐posterior laxity after sequential sectioning of the PCL and MFLs. The knees underwent anterior‐posterior drawer testing at various flexion angles with 100 N force. Results showed the MFLs contributed 71% to restraining PTT in PCL‐deficient knees at 90° flexion, and 28% in PCL‐intact knees [19]. In contrast, the present study found no significant role of the MFLs in restraining PTT.

There are important differences between the cited study and the present setup. First, in this study the PCL was cut before the MFL [19]. Second, the study used a test setup with 4 degrees of freedom versus 6 degrees of freedom in the setup used in the present study. Another key difference is the force‐controlled movement compared to the position‐controlled movement used in the present study. There is more wear out of the ligamentous structures using a force‐controlled movement in the different cutting states because the maximum force is reached in every movement and therefore the PTT might increase over the cutting steps. Also, the deduction of the involvement of the MFLs from the PCL‐deficient state to the PCL‐intact state using load‐displacement curves in a force‐controlled setup, as performed in this study, therefore does not appear to be validly possible based on our investigations.

Another biomechanical study tested 14 human cadaveric knees in a material testing machine to determine the structure responsible for a decreased posterior drawer in the PCL‐deficient knee in internal rotation compared to neutral rotation [29]. In this study the MFLs were cut prior to the PCL, and the posterior drawer was tested in neutral and internal tibial rotation. A force‐controlled movement was performed, the force used to perform a PTT is not reported. It showed no influence of the MFLs to the extent of the posterior drawer in the PCL‐intact knee in neutral tibial rotation as well as in internal tibial rotation.

This again confirms the differences between the role of the MFLs in the PCL‐deficient and the PCL intact knee. The MFLs seem to act as a secondary restraint to PTT. However, in our study using the method of superposition the cutting order should be a less decisive influence on the measured contribution of the cut structures compared to a force‐controlled setup and measuring of the increase of PTT.

Supporting the thesis of an influence of the MFL in restraining PTT, a study group tested ten porcine knees in a 6‐DOF robotic testing setup. This study showed a significant increase in PTT after cutting the PCL and another significant increase of PTT after additional cutting the MFL. It demonstrated the function of the porcine MFL as a secondary restraint to PTT. The anatomy of the porcine MFL differs from human knees as there only exists one MFL which passes approximately like the human pMFL [20]. A transfer to human biomechanics cannot be assumed. Nevertheless, the influence of MFLs to the stability of the porcine knee should be considered in further biomechanical studies using porcine models.

Regarding the prevalence of the MFLs an aMFL was found in 81% of the knees tested. We thus saw an above‐average prevalence of the aMFL, which is described in a systematic review as 40.1% [6]. According to the review, a pMFL is found in 60.9% of knee joints [6]. The knee joints examined in this study also exhibited a pMFL slightly more frequently than described on average with 69% of the knees tested. The presence of both MFLs is described as rather rare at 17.6% [6]. Here a substantially higher prevalence of 63% of cases was found. A possible reason might be that in the cited review MRI studies, arthroscopic studies as well as cadaveric studies were included to describe the prevalence of MFLs [6].

The role of the PCL in restricting PTT of the knee is described by several authors. Early studies show an increasing influence of the PCL at higher degrees of flexion [2, 16, 18, 28]. Near extension, posterolateral structures act as important inhibitors of PTT [16, 18, 28].

In the present study, we could show similar results as cutting of the PCL reduced the force needed to reach the PTT of the intact state primarily in higher flexion angles throughout all rotation states. Near extension there was shown a smaller influence of the PCL in limiting PTT. This effect was more evident in internal and external rotation than in the neutral rotational state. This can be explained by the involvement of the posterolateral structures like the lateral collateral ligament, the arcuate complex and the popliteal tendon mainly in external rotation, but also in neutral rotation [2, 16, 18, 28]. In internal tibial rotation this effect is caused by the involvement of the posteromedial capsule and the superficial medial collateral ligament in restraining PTT [29].

The present study is of clinical relevance, due to the implications for clinical practice. According the aforementioned studies, a dissection of the MFLs to gain access to the PCL during reconstruction surgery would lead to increased posterior instability [19]. In contrast the present study showed that the MFLs do not restrain against a PTT in the PCL‐intact knee. Assuming that a PCL‐reconstructed knee shows similar kinematics compared to a PCL‐intact knee it can be considered that sparing, or reconstruction of the MFLs, is not needed to restore knee kinematics in the PCL‐deficient knee. However, it must be taken in account that the MFLs might contribute to the function of the lateral meniscus [12, 27]. A cadaveric biomechanical study investigating the effect of the MFLs on the motion of the lateral meniscus during knee motion could show that the MFLs, when tensioned, cause a medial, superior and anterior displacement of the posterior horn of the lateral meniscus. By this the congruity of the femoral condyle and the lateral meniscus was increased [27]. Also, tears of the posterior horn of the lateral meniscus only led to increased tibiofemoral contact pressures if the MFLs were injured at the same time [1, 11, 15]. Therefore, a resection of the MFLs, if not necessary, should be avoided [12].

There are several limitations to this study as we performed testing on human cadaveric knees. The forces of muscle tension affecting the knee kinematics as well as kinematic effects of the skin removal could not be measured. The age of the body donors (mean age 67.3 ± 7.4 years) also is not representative of the patient collective typically undergoing arthroscopic joint surgery.

Also, due to normal human variability, only 10 of the 16 knees presented both MFLs. However, power analysis indicated that this number of subjects was sufficient for this study. Another limitation to this study is that only native kinematics of the knee were tested and a possible wear out of the remaining restraints of PTT after the cutting steps cannot be taken into consideration during testing of PCL and MFL‐deficient states. This is necessary in a position‐controlled setup to validly measure the reduction of force after the different cutting steps.

## CONCLUSION

The MFLs do not contribute to restriction of a PTT in any flexion angle, while the PCL acts as the primary restraint against PTT from 0°–90° knee flexion. This effect was seen in neutral rotation as well as in tibial internal and external rotation. Our findings indicate that a resection of the MFLs during PCL reconstruction surgery does not destabilise the knee.

## AUTHOR CONTRIBUTIONS

**Lucas K. Palma Kries:** Writing; statistical analysis; proofreading. **Wenke Liu:** Testing and data acquisition. **Michael J. Raschke:** Supervision; internal review; proofreading. **Alina Albert:** Testing and data acquisition. **Christian Peez:** Internal review; proofreading. **Elmar Herbst:** Internal review; proofreading. **Christoph Kittl:** Internal review; proofreading. **Adrian Deichsel:** Conception and design; testing and data acquisition; statistical analysis; proofreading.

## CONFLICT OF INTEREST STATEMENT

Elmar Herbst is Deputy Editor‐in‐Chief for the Knee Surgery, Sports Traumatology and Arthroscopy (KSSTA). Adrian Deichsel is Web Editor for the Knee Surgery, Sports Traumatology and Arthroscopy (KSSTA). All other authors declare no conflicts of interest.

## ETHICS STATEMENT

The specimens were dissected and biomechanically tested under the approval of the Institutional Ethics Committee of the University of Muenster (File number 2023‐407‐f‐S).

## Data Availability

Data is available from the corresponding author upon reasonable request.
